# Determinants of Maternal RSV Vaccination Uptake: A Narrative Review

**DOI:** 10.3390/vaccines14040293

**Published:** 2026-03-26

**Authors:** Aikaterini I. Nikolaou, Alexandra Soldatou, Georgia-Christiana Grantzi, Vasileios Giapros, Fani Ladomenou

**Affiliations:** 1 Department of Pediatrics, School of Medicine, University of Ioannina, 45500 Ioannina, Greece; nikaikaterini@gmail.com; 2 Neonatal Intensive Care Unit, School of Medicine, University of Ioannina, 45500 Ioannina, Greece; vgiapros@uoi.gr; 3 Department of Pediatrics, School of Medicine, National and Kapodistrian University of Athens, 15772 Athens, Greece; alsoldat@med.uoa.gr; 4 Evangelismos General Hospital, 10676 Athens, Greece; ginagrantzi55@hotmail.com

**Keywords:** respiratory syncytial virus, maternal vaccination, pregnancy, vaccine uptake, vaccine hesitancy, monoclonal antibodies, health services accessibility, health equity

## Abstract

Maternal vaccination against respiratory syncytial virus (RSV) represents a major advance in early-life infection prevention. Although clinical efficacy and early real-world effectiveness are well established, sustained population-level impact depends on equitable uptake. This review synthesizes determinants influencing maternal RSV vaccination within the evolving dual-strategy landscape that includes both maternal vaccination and infant monoclonal antibody prophylaxis. A structured narrative review was conducted following PRISMA principles. PubMed/MEDLINE and Google Scholar were searched for studies published between January 2022 and February 2026. Eligible studies examined behavioral, interpersonal, structural, economic, and policy determinants of maternal RSV vaccination uptake, as well as early implementation and modelling evidence. Findings were integrated within a multilevel analytical framework. Maternal uptake is shaped by interacting determinants across individual, healthcare provider, and health system domains. Key drivers include perceived infant disease severity, vaccine safety confidence, perceived effectiveness, and prior antenatal vaccination behavior. Healthcare provider recommendation consistently emerges as the strongest facilitator. Coverage variability reflects differences in reimbursement, antenatal care integration, and national policy endorsement. The coexistence of maternal vaccination and infant monoclonal antibody strategies introduces additional comparative decision-making complexity. Early implementation data indicate heterogeneous uptake and socioeconomic gradients, while modelling demonstrates sensitivity to coverage, timing, epidemiology, and cost. Translating biological efficacy into sustained public health benefit requires coordinated behavioral, structural, and policy strategies, strong provider engagement, and context-sensitive implementation frameworks to ensure equitable coverage.

## 1. Introduction

Respiratory syncytial virus (RSV) is a major global cause of acute lower respiratory tract infection (LRTI) across the life course, affecting both young children and older adults [1,2,3]. While RSV remains a leading cause of hospitalization and healthcare utilization in infants—particularly during the first six months of life [1]—it is increasingly recognized as a significant contributor to morbidity and healthcare burden among older adults and individuals with underlying conditions [2,3]. This expanded epidemiological understanding has driven the recent development and implementation of RSV vaccination strategies targeting older adult populations in multiple countries [2,3]. Within this broader public health context, prevention of RSV in early infancy remains a critical priority, given the heightened vulnerability of neonates, the concentration of severe disease in the first months of life, and the limited availability of therapeutic options [1]. Maternal immunization has therefore emerged as a key preventive strategy, aiming to bridge this early-life vulnerability through transplacental transfer of protective antibodies and to complement existing RSV prevention approaches across the life course [4,5].

The greatest burden occurs during the first six months of life, when immunological immaturity and limited therapeutic options increase vulnerability to severe disease [1]. RSV-associated hospitalization rates among otherwise healthy term infants remain substantial, with seasonal surges placing additional strain on healthcare systems [6,7] and generating considerable indirect societal costs, including parental work absenteeism [6]. In high-burden settings, RSV also contributes to infant mortality; modelling from South Africa suggests that maternal vaccination could avert substantial deaths under diverse epidemiological scenarios, with projected benefits outweighing potential risks [8].

Maternal vaccination is biologically supported by the transplacental transfer of immunoglobulin G, offering passive protection during the period when neonatal immune responses are still developing [4]. Empirical evidence confirms both the efficiency of this transfer and the persistence of RSV-specific antibodies in early infancy [5]. In the wake of post-pandemic disruptions to RSV transmission, resulting shifts in seasonal dynamics and population susceptibility further highlight the urgency of implementing scalable preventive strategies for this vulnerable age group [9].

Recent advances in structural vaccinology and monoclonal antibody development have reshaped RSV prevention [10,11]. Evidence from a pivotal phase 3 randomized controlled trial demonstrated that maternal RSVpreF vaccination significantly reduced medically attended RSV-associated LRTI and hospitalization in early infancy [12], while long-acting monoclonal antibody prophylaxis, including nirsevimab, achieved comparable efficacy among late-preterm and term infants [13]. These findings are reinforced by pooled evidence from systematic reviews, meta-analyses, Cochrane syntheses, and regulatory assessments, all of which confirm robust immunogenicity and favorable efficacy and safety profiles without increased perinatal risk [14,15,16]. Real-world obstetric data from routine immunization programs likewise show no increased risk of preterm birth or adverse maternal or neonatal outcomes [17,18].

Early real-world effectiveness data corroborate trial findings. Test-negative and multicenter analyses from Argentina and the United Kingdom demonstrated substantial reductions in RSV-associated hospitalization among infants born to vaccinated mothers [19,20], while post-implementation surveillance from the United States and Italy suggests measurable declines in RSV-related hospitalization following deployment of maternal vaccination and/or monoclonal antibody programs [21,22]. Modelling and economic evaluations indicate that projected public health impact depends critically on coverage levels, timing, epidemiological context, and cost structures [23,24,25,26]. National guidance documents increasingly integrate these preventive strategies into RSV management frameworks [27,28], while recent syntheses situate RSV vaccination alongside influenza and COVID-19 immunization within routine RSV prevention strategies [29].

Nevertheless, biological efficacy does not automatically translate into population-level impact. Maternal vaccination programs historically demonstrate variable coverage across settings, shaped by interacting individual, interpersonal, structural, economic, and policy determinants. Emerging behavioral evidence from Europe, North America, and Asia indicates heterogeneous willingness to accept maternal RSV vaccination, influenced by perceived infant risk, safety concerns, prior vaccination behavior, healthcare provider recommendation, and comparative evaluation of infant monoclonal antibody alternatives [30,31,32,33,34,35,36,37]. The concurrent availability of maternal vaccination and infant monoclonal antibody prophylaxis further introduces a novel comparative dimension into antenatal decision-making [32,34].

To date, existing reviews on RSV prevention have largely focused on isolated aspects of vaccine acceptance or implementation, without integrating the full spectrum of determinants that shape real-world uptake. In particular, prior syntheses have not systematically combined behavioral, interpersonal, structural, economic, and policy-level factors within a unified analytical framework, nor have they examined maternal RSV vaccination within the context of the emerging dual-strategy prevention landscape that includes both maternal immunization and infant monoclonal antibody prophylaxis.

This narrative review addresses these gaps by synthesizing current evidence on determinants influencing maternal RSV vaccination uptake and delineating its added value in three key respects. First, it provides a temporally updated synthesis of evidence emerging from the early implementation phase of both maternal RSV vaccination and long-acting monoclonal antibody strategies. Second, it advances an integrative, multilevel analytical framework that systematically incorporates behavioral, interpersonal, structural, economic, and policy determinants, thereby enabling a more comprehensive understanding of the mechanisms shaping real-world uptake. Third, it explicitly situates maternal vaccination within the evolving dual-strategy RSV prevention landscape, incorporating the comparative and potentially substitutive or complementary dynamics between maternal immunization and infant monoclonal antibody prophylaxis. In this context, the coexistence of these strategies creates a comparative decision-making environment in which evaluation between alternative preventive options constitutes an important determinant of maternal vaccination uptake. Collectively, this approach offers a conceptually coherent and implementation-oriented perspective that extends beyond prior literature, which has predominantly examined individual determinants or isolated dimensions of RSV prevention strategies. Importantly, by integrating multilevel determinants within a dual-strategy prevention context and incorporating emerging real-world and behavioral evidence, this review provides a structured and practice-oriented synthesis that extends beyond prior descriptive summaries.

## 2. Methods

This narrative review was conducted using a structured literature search informed by selected PRISMA (Preferred Reporting Items for Systematic Reviews and Meta-Analyses) principles, with emphasis on transparency in study identification, screening, and reporting. As a narrative synthesis aiming to integrate heterogeneous evidence—including behavioral studies, qualitative research, real-world implementation analyses, surveillance data, and economic modelling—a full systematic review methodology and complete adherence to PRISMA guidelines were not considered appropriate. Accordingly, PRISMA-aligned elements were applied to the literature search, study selection process, and reporting of results, while protocol registration, dual independent screening, and formal risk-of-bias assessment were not undertaken.

PubMed/MEDLINE was used as the primary database for literature retrieval. In addition, targeted searches of Google Scholar were conducted to identify relevant grey literature and policy documents, rather than to perform a fully reproducible systematic search. The search covered studies published between January 2022 and February 2026, reflecting the period during which maternal RSV vaccination and long-acting monoclonal antibodies became clinically and policy relevant. The search strategy combined controlled vocabulary terms and free-text keywords related to respiratory syncytial virus (RSV), maternal vaccination, pregnancy, vaccine uptake, acceptance, intention, vaccine hesitancy, determinants, implementation, monoclonal antibodies, and nirsevimab, using Boolean operators (AND/OR) as appropriate. Database-specific adaptations of the search strategy were applied. Grey literature and policy documents were additionally identified through targeted searches of public health agency websites, including the World Health Organization. Detailed search strategies, including full search strings, Boolean operators, and applied limits, are provided in Supplementary Table S1. The absence of additional databases, such as Embase, is acknowledged as a limitation.

**Figure 1:**
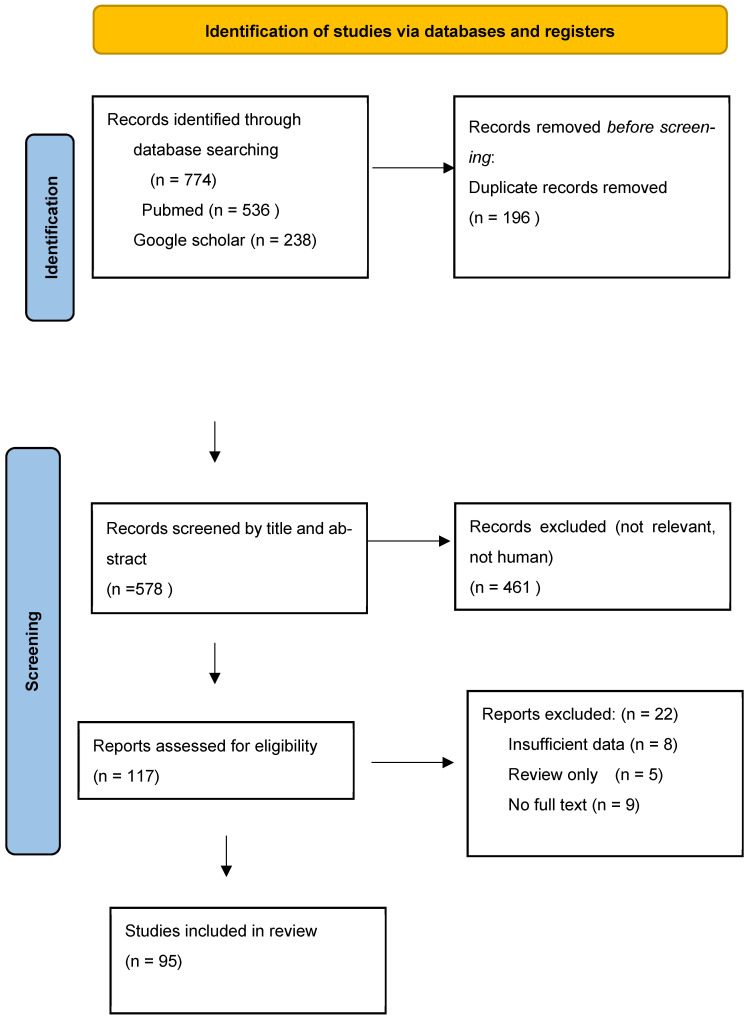
PRISMA flow diagram of search procedure.

Following duplicate removal, records were screened in two sequential stages. First, titles and abstracts were reviewed to exclude clearly irrelevant studies. Second, full-text articles were assessed for eligibility based on predefined inclusion and exclusion criteria. Studies were eligible for inclusion if they addressed determinants of maternal RSV vaccination uptake, parental preferences between preventive strategies, real-world implementation data, economic or modelling analyses, or policy-relevant contextual factors. Randomized controlled trials evaluating vaccine efficacy were included selectively for contextual interpretation but were not the primary focus of behavioral synthesis. Studies were excluded if they were unrelated to RSV prevention, limited to preclinical or immunogenicity-only outcomes without behavioral or implementation relevance, or lacked sufficient empirical data, unless they provided substantive policy insight. Study selection decisions were reviewed by the authors, and discrepancies were resolved through discussion. The study selection process is illustrated in Figure 1.

The diversity of study designs, populations, and outcome definitions precluded meaningful quantitative pooling of results; therefore, quantitative meta-analysis was not undertaken. Findings were synthesized narratively and organized thematically across behavioral, structural, economic, and policy-level determinants within a multilevel analytical framework. Given the narrative design and methodological heterogeneity, formal risk-of-bias tools were not applied. However, key quality features—including study design, sample size, clarity of outcome definitions, and potential sources of bias—were considered when interpreting the strength and consistency of the evidence. A summary of included representative empirical studies is provided in Table 1. A complete list of included studies is provided in Supplementary Table S2. While a formal systematic review methodology was not applied, efforts were made to ensure a comprehensive and balanced synthesis through structured search strategies, predefined inclusion criteria, and careful consideration of study characteristics and potential sources of bias during data interpretation.

## 3. Epidemiological and Clinical Context

RSV remains a leading cause of hospitalization among infants globally, particularly during the first six months of life [1]. Surveillance data from diverse regions—including Europe—consistently demonstrate substantial early-life morbidity and healthcare utilization [6], while seasonal peaks continue to exert pressure on pediatric services [7].

Post-pandemic disruptions in viral transmission altered traditional RSV seasonality, producing atypical resurgences and increased susceptibility among previously unexposed cohorts [9]. These shifts have reinforced the need for preventive strategies capable of protecting infants during the period of highest vulnerability to severe LRTI.

The biological rationale for maternal RSV vaccination is grounded in maternal–fetal immune mechanisms. Transplacental transfer of maternal immunoglobulin G provides passive neonatal protection during early infancy, when endogenous immune responses are immature [4]. Empirical evidence confirms efficient antibody transfer and early-life persistence of RSV-specific antibodies [5], although physiological antibody decline limits the duration of passive protection and highlights the need for optimally timed antenatal vaccination [5,43]. In addition to pathogen-specific benefits, maternal vaccination may also reduce neonatal infections and subsequent antibiotic exposure, aligning with broader antimicrobial stewardship goals [44].

Consistent with accumulated clinical and post-licensure evidence, randomized trials, pooled analyses, and real-world effectiveness studies demonstrate substantial protection against RSV-associated lower respiratory tract infection and hospitalization in early infancy following maternal RSVpreF vaccination and long-acting monoclonal antibody administration, including nirsevimab [2,12,13,14,19,20,44,45,46,47,48,49]. Additional post-licensure pharmacovigilance and obstetric data have not identified significant safety concerns [14,17,18,45,50], and early programmatic implementation has been associated with measurable reductions in RSV-related hospitalizations in several settings [21,22].

Despite strong clinical and effectiveness evidence, translation into sustained public health impact depends on implementation performance and equitable maternal uptake. Durable reductions in RSV morbidity depend on timely administration and adequate, equitable uptake of maternal vaccination during pregnancy. Understanding determinants of acceptance, access, and coverage is therefore essential to bridging the gap between clinical efficacy and real-world public health benefit.

## 4. Determinants of Maternal RSV Vaccination Uptake

Maternal uptake of RSV vaccination is shaped by interacting determinants across individual, interpersonal, structural, and policy domains [30,31,32,33,34,35,36,51,52], summarized in Table 2. Importantly, the relative influence of these determinants is not uniform, with healthcare provider recommendation, safety confidence, and perceived infant risk emerging as consistently dominant drivers across studies. In this review, behavioral (individual-level) determinants refer to maternal perceptions, beliefs, and attitudes influencing vaccination intention; interpersonal determinants relate to interactions with healthcare providers and social influences; structural determinants encompass health system characteristics such as access, reimbursement, and service delivery; and policy-level determinants include national recommendations, regulatory frameworks, and institutional endorsement. These categories reflect distinct but interacting levels of influence, ranging from individual cognitive drivers to system-level and policy determinants that shape implementation [53,54,55].

While robust clinical efficacy and post licensure safety data provide the foundation for program implementation [2,12,19,20,50], maternal decision making remains inherently multidimensional, shaped by risk perception, trust, access, and contextual influences. The multilevel framework adopted in this review aligns with established implementation models such as CFIR and the Theoretical Domains Framework [53,54,55], strengthening conceptual coherence and facilitating the translation of identified determinants into targeted implementation strategies.

### 4.1. Distinguishing Intention from Observed Uptake

Intention and preference studies rely on hypothetical scenarios and may not directly translate into real-world behavior. In contrast, observed uptake reflects the influence of additional structural, financial, and service delivery factors that shape implementation under routine conditions. For this reason, intention and preference findings are interpreted separately from observed coverage data throughout this review, in line with established distinctions in behavioral and implementation research.

### 4.2. Determinants of Vaccination Intention

Determinants of vaccination intention primarily reflect behavioral and cognitive processes, including risk perception, safety concerns, and perceived benefits, which shape hypothetical acceptance under anticipated conditions rather than actual vaccination behavior.

**Perceived Infant Risk and Disease Severity**

Reported intention to accept maternal RSV vaccination varies across settings. In an England-wide survey, 89.5% of respondents reported willingness to receive maternal RSV vaccination, indicating high baseline acceptance under hypothetical conditions [31]. Perceived susceptibility to and severity of RSV infection in early infancy consistently predict maternal intention to vaccinate [30,32,35]. In a Greek cohort, heightened perception of disease severity and infant vulnerability was associated with greater acceptance of maternal vaccination [30], with similar associations observed in Italy and North America, where recognition of RSV as a significant cause of infant hospitalization increased willingness to vaccinate [32,35].

Baseline awareness and perceived risk of RSV vary across populations [31,33]. Higher perceived likelihood of infant RSV illness and positive vaccine attitudes have been consistently associated with greater willingness to accept maternal vaccination and infant monoclonal antibodies [33], underscoring the importance of risk communication within implementation strategies. Qualitative data from Australia show that mothers seek clear, consistent, provider-endorsed information, and that informational gaps may delay decision-making [56].

Evidence from Asia aligns with these findings. In Taiwan, greater RSV knowledge and higher perceived risk were associated with increased willingness to accept infant RSV vaccination [37]; in Japan, knowledge of disease and vaccine characteristics correlated with favorable attitudes [57]; and in Turkey, greater awareness was linked to more positive views toward immunization [58]. Collectively, these studies underscore that informational deficits and perceived disease risk represent consistent—and modifiable—determinants of maternal decision-making.

**Perceived Vaccine Effectiveness**

Perceived effectiveness is closely tied to risk appraisal. Survey data indicate that higher perceived likelihood of infant RSV illness and positive vaccine attitudes are associated with greater willingness to accept maternal RSV vaccination and infant monoclonal antibodies [33]. While the behavioral impact of communicating real-world effectiveness data has not been directly quantified, the association between perceived benefit and intention suggests that evidence of vaccine effectiveness may play an important role in shaping maternal decision-making [32,33,48,50].

**Safety Concerns During Pregnancy**

Safety concerns remain the most consistently reported barrier to maternal RSV vaccine acceptance [31,34]. In England, uncertainty regarding vaccine safety was the principal driver of hesitancy [31], while Canadian respondents with safety reservations were more likely to prefer infant monoclonal antibody strategies [34].

Accumulating evidence does not indicate major safety signals: meta-analyses and obstetric cohort studies demonstrate robust immunogenicity without increased perinatal risk [14,15,17,59]. Nonetheless, hesitancy persists, highlighting divergence between objective safety evidence and subjective risk perception [60]. Broader analyses situate vaccine decisions within dynamics of institutional trust and communication [60]. Transparent, dialogue-based counselling remains central, while digital discourse analyses reveal recurring safety narratives and trust-related concerns influencing maternal appraisal [61].

**Prior Maternal Vaccination Behavior**

Previous acceptance of pertussis or influenza vaccination predicts willingness to receive the RSV vaccine [30,32,33,36]. These decisions are embedded within broader antenatal immunization culture, with variations influenced by provider recommendation and healthcare setting practices [62].

**Comparative Evaluation of Preventive Strategies as a Determinant of Maternal Uptake**

A distinctive feature of RSV prevention is the simultaneous availability of maternal vaccination and infant monoclonal antibody prophylaxis, creating a comparative decision environment [32]. Importantly, this comparative evaluation constitutes a key behavioral determinant of maternal vaccination uptake, as decisions are made between alternative preventive options rather than in isolation.

Data from Canada (COVERED Study) illustrate this pattern, with 77% of participants indicating willingness to accept maternal RSV vaccination compared with 55% for infant monoclonal antibodies, and 79% expressing a preference for maternal vaccination [34]. Despite overall high acceptance of both modalities, preference may shift toward infant monoclonal antibodies when concerns regarding vaccination during pregnancy are present [34]. Preferences are shaped by perceived maternal safety, directness of infant protection, trust in healthcare providers, and prior antenatal vaccination experience.

Comparable patterns have been observed in the United Kingdom, where safety perceptions and trusted healthcare advice emerged as principal determinants of preference [63]. Additional survey data confirm that perceptions of maternal safety, direct infant protection, and institutional trust influence acceptance and comparative considerations [38,64], while perspectives from immigrant communities highlight further cultural and communication dimensions [65]. Economic modelling suggests that perceived trade-offs between intervention characteristics may also influence stated preferences [66,67].

These findings demonstrate that maternal vaccination intention is shaped within a comparative decision-making context involving parallel preventive options, and that such comparative processes directly influence potential uptake [32,34,38]. Importantly, these strategies are delivered through distinct clinical and organizational pathways, with maternal vaccination integrated into antenatal care services and monoclonal antibody prophylaxis implemented through neonatal and paediatric care settings [40,51]. These approaches differ in timing, target population, clinical indication, and reimbursement and delivery structures within healthcare systems [23,24,25,26,66], highlighting the role of structural and behavioral distinctions in shaping maternal preferences and informing the comparative framework summarized in Table 3 [39,51].

**Interpersonal Determinants: Healthcare Provider Influence**

Healthcare provider recommendation is one of the most consistently identified facilitators of maternal RSV vaccine acceptance [30,31,34,36]. In the England-wide survey, willingness to receive antenatal RSV vaccination increased substantially when recommended by a trusted healthcare professional [31]. Comparable findings were observed in Greece and Canada, where endorsement from obstetricians or midwives significantly influenced intention [30,34].

Mixed-methods evidence suggests that the strength, clarity, and timing of provider recommendation are critical factors [36]. Provider communication serves as a bridge between scientific evidence and maternal decision-making, mediating interpretation of safety and effectiveness data.

These findings are consistent with established patterns observed in other antenatal vaccination programs, reinforcing the central role of obstetric care providers in promoting vaccination intention and acceptance.

**Sociodemographic and Contextual Determinants**

Associations between sociodemographic characteristics and maternal RSV vaccine acceptance appear context-dependent. Education level has been associated with increased willingness in some studies, potentially reflecting differences in health literacy and access to information [30,35]. However, effect sizes and consistency vary across populations.

Cross-national differences in intention and preference further suggest the influence of contextual factors, including institutional trust, prior experience with antenatal vaccination campaigns, and broader cultural attitudes toward vaccination [30,31,34,35].

### 4.3. Determinants of Observed Uptake

Vaccination uptake reflects the translation of intention into behavior and is conceptually distinct from stated intention, as it represents realized behavior under real-world conditions. It is shaped by structural and system-level determinants, including healthcare access, service organization, reimbursement policies, and delivery infrastructure, which collectively determine the feasibility of translating intention into actual vaccination behavior. Real-world data from a US cohort study reported maternal RSV vaccination uptake of 64.0% among eligible pregnant individuals, with 70.1% of eligible neonates receiving nirsevimab and overall RSV protection exceeding 80% during most of the study period [68].

**Health System and Structural Determinants**

Real-world data demonstrate variability in maternal RSV vaccination uptake across healthcare systems [52]. Differences in national policy endorsement, reimbursement mechanisms, integration into antenatal care pathways, and logistical accessibility appear to shape program performance. Implementation contexts characterized by clear recommendations and coordinated delivery structures tend to exhibit more consistent uptake, whereas fragmented systems may experience slower or uneven adoption [51,52].

Professional society endorsement constitutes an additional structural determinant influencing program implementation. A recent position statement from the Mexican Association of Pediatrics outlining recommendations for immunoprevention of RSV during pregnancy and infancy reflects growing institutional support for maternal vaccination strategies within Latin America [29]. Such national-level guidance may shape provider recommendation behavior, facilitate policy alignment, and promote integration of maternal RSV vaccination into routine antenatal care services.

Programmatic infrastructure and financing mechanisms can also directly influence access to RSV preventive products. In the United States, expansion of birthing hospital enrollment in the Vaccines for Children program was implemented to facilitate infant immunization against RSV and reduce structural barriers at the point of care [69]. This example illustrates how policy instruments and funding frameworks can enhance equitable access, particularly for socioeconomically vulnerable populations, and demonstrates the role of system-level interventions in translating recommendations into practice.

Discrete choice experiment findings from The Netherlands indicate that cost, convenience, and delivery setting significantly influence vaccination uptake and implementation [52]. These results suggest that structural accessibility—including financial coverage, integration into routine antenatal visits, and minimization of additional appointments—plays a critical role in translating intention into actual uptake.

Nationwide survey data from Japan further illustrate how structural and policy contexts shape coverage [39]. In that setting, maternal RSV vaccine uptake was influenced not only by individual-level attitudes but also by healthcare system factors such as access pathways and provider engagement. This underscores that behavioral determinants operate within broader organizational frameworks that can either facilitate or constrain vaccination. Complementary qualitative evidence highlights additional barriers unique to the Japanese context, including limited public awareness of maternal RSV vaccination, insufficient provider recommendation, and uncertainty regarding reimbursement mechanisms [70]. These findings emphasize that even in health systems with established antenatal care infrastructure, information gaps and policy ambiguity can hinder program uptake. Broader regional commentaries further contextualize these structural challenges. In Southern Europe, strengthening routine antenatal care and integrating maternal vaccination within established obstetric services have been identified as key strategies to close the maternal vaccination gap and ensure equitable implementation of new RSV prevention programs [40].

When maternal RSV vaccination is embedded within established antenatal vaccination programs, operational barriers may be reduced and provider recommendation more consistently implemented. Conversely, unclear reimbursement policies, supply instability, or limited integration into routine antenatal pathways may constrain program performance despite generally favorable maternal attitudes. These structural conditions therefore influence the degree to which individual intention can translate into sustained and equitable program delivery. The multilevel framework presented here reflects an integrative conceptual synthesis derived from convergent behavioral and implementation evidence rather than a single predefined theoretical model.

**Equity and Sociodemographic Disparities in Uptake**

Although preliminary analyses suggest potential disparities, comprehensive population-level equity assessments of observed maternal RSV vaccine uptake remain limited. Early variability in coverage across implementation settings indicates that structural and socioeconomic factors may influence access to and uptake of vaccination [51].

### 4.4. Real-World Uptake and Early Implementation Signals

Despite established clinical efficacy and early real-world effectiveness [12,19,20], observed program coverage remains heterogeneous [51]. A recent systematic review and meta-analysis identified marked cross-national variability in uptake, reflecting differences in policy endorsement, reimbursement mechanisms, and delivery structures [51]. National data from Japan demonstrated measurable but variable maternal vaccine coverage shaped by perceived infant risk, provider recommendation, and safety confidence [39]. Similar patterns emerged in the United States, where a multicentre cohort reported concurrent RSVpreF and nirsevimab uptake with modest overall coverage, gradual seasonal increases, and persistent sociodemographic disparities [68]. Provider preparedness further influenced implementation: surveys of Turkish pediatricians revealed variability in familiarity with RSV prevention strategies [71], while US physicians’ perceptions of disease burden and preventive preferences affected counselling practices [72].

Socioeconomic gradients also modulate coverage. In France, adherence to the nirsevimab campaign was associated with sociodemographic and healthcare access variables [41], and early UK data showed comparable disparities without excess adverse obstetric outcomes [42]. US surveillance during the 2023–2024 season documented expanding but incomplete infant protection through maternal vaccination and/or nirsevimab [73], with additional state-level and Vaccine Safety Datalink analyses confirming site-level and demographic variability in uptake [74,75,76].

In Europe, Austrian real-world data linked uptake of RSVpreF vaccination and nirsevimab with reductions in RSV disease burden [77], and surveillance in the United States and Italy similarly documented post-implementation declines in RSV-associated hospitalizations [21,22,78]. Although bronchiolitis admissions are not exclusively RSV-related, these convergent findings suggest that coordinated deployment can reduce seasonal pediatric respiratory morbidity. Accurate estimation of maternal vaccine coverage depends on surveillance quality; Australian data highlighting discrepancies between registry-based and administrative reporting underscore the importance of robust data linkage and completeness [79].

Overall, structured and coordinated implementation can translate demonstrated biological efficacy into measurable population-level benefit. However, much of the behavioral literature continues to assess stated intention rather than verified vaccination behavior [30,31,34], underscoring the need to integrate behavioral insights with implementation data to clarify how determinants operate under routine program conditions.

## 5. Cross-Cutting Themes and Research Gaps

Several cross-cutting themes emerge from the current evidence base. First, the distinction between vaccination intention and observed uptake remains insufficiently characterized. Although high levels of stated willingness have been reported—reaching 89.5% in UK survey data—real-world uptake remains more variable across settings, highlighting a persistent gap between intention and observed vaccination behavior [31,51,68]. This divergence highlights a persistent gap between intention and uptake, underscoring the need for implementation-focused research. Overall, while several determinants are recurrent across settings, their relative impact is shaped by the interaction between behavioral, structural, and policy-level factors within specific implementation contexts. Most behavioral studies rely on cross-sectional designs assessing hypothetical acceptance rather than longitudinal vaccination behavior, limiting causal inference regarding determinants of actual uptake. Broader maternal vaccination research similarly identifies gaps in understanding how behavioral drivers interact with structural and policy-level factors during pregnancy [80]. Addressing these gaps requires prospective, registry-linked cohort studies capable of examining how intention translates into uptake, supported by large-scale data linkage for accurate coverage and outcome assessment [81].

Second, comparative decision-making now defines the RSV prevention landscape. The coexistence of maternal vaccination and infant monoclonal antibody prophylaxis creates a dual-strategy framework distinct from traditional antenatal programs, with maternal vaccination providing transplacental protection and monoclonal antibodies offering direct infant protection [32,34,82]. Policy developments—including ACIP recommendations for clesrovimab in the United States [83] and European endorsement of maternal RSV vaccination [84]—reflect increasing institutional integration. At the individual level, preferences are shaped by safety perceptions, perceived control, and timing of protection, reflecting decision-making processes that may not translate into real-world uptake, while modelling shows that population-level impact depends on coverage, timing, and implementation efficiency [23].

From a health system perspective, these strategies operate through parallel but distinct implementation pathways, with maternal vaccination delivered within antenatal care services and monoclonal antibodies administered through neonatal or paediatric platforms [32,34,51]. This separation has implications for program design, resource allocation, reimbursement structures, and workforce coordination [23,24,25,26,66,67], underscoring the need for integrated but pathway-specific implementation strategies and reinforcing the distinction between stated preferences and observed uptake [39,51].

Economic evaluations demonstrate strong context sensitivity, with cost-effectiveness varying according to epidemiology, pricing, health system capacity, financing, and achievable coverage across settings [24,25,26,66,85,86]. Comparative modelling from Canada and the United States further shows that projected impact depends on product characteristics, baseline disease burden, and coverage assumptions [87,88,89], while a systematic review highlights substantial heterogeneity in modelling approaches [90].

Epidemiological modelling emphasizes the importance of temporal alignment. Regional variability in RSV seasonality influences optimal intervention timing [91], and alignment with local transmission dynamics significantly affects projected effectiveness and cost-efficiency [92]. Dynamic models further highlight sensitivity to coverage, duration of protection, and baseline epidemiology [93], indicating that optimization requires integration of epidemiological, economic, and health system factors.

Third, the translation of safety and effectiveness evidence into behavioral change remains incompletely understood. Although post-marketing surveillance, pooled analyses, and observational data have not identified unexpected safety signals [2,14,17,50], safety concerns persist [31,34]. Evidence on how communication of safety and real-world effectiveness influences uptake remains limited, and experimental or implementation-focused studies are scarce. Consequently, the mechanisms through which updated evidence shapes vaccination behavior remain underexplored, particularly regarding the transition from intention to uptake.

Digital information environments may further influence maternal risk appraisal. Social media analyses from Italy identify recurring safety narratives, institutional trust dynamics, and comparative framing of preventive options during early rollout [61], suggesting that public discourse may influence decision-making alongside clinical counselling.

Fourth, generalizability is constrained by the concentration of behavioral evidence in high-income settings. Although modelling studies expand geographic representation [24,25,26], substantial gaps remain in context-specific behavioral and implementation data. Additional modelling studies highlight sensitivity to epidemiology and timing across settings [52,85,86,89,91,93]. Given the global RSV burden [1], context-specific research is essential to support equitable and effective implementation.

## 6. Equity and Ethical Considerations

Equitable implementation of maternal RSV vaccination requires addressing both structural access barriers and the conditions that support informed, autonomous decision-making. While higher education and health literacy have been associated with increased intention to vaccinate [30,35], systematic analyses of inequities in observed coverage and real-world uptake remain limited. Available evidence indicates that reimbursement mechanisms, policy endorsement, and integration into routine antenatal care shape differential access to and uptake of vaccination across settings [51]. Equity concerns are particularly salient given the disproportionate RSV burden in low- and middle-income countries [1]. The vast majority of global RSV-related deaths occur in LMICs, making equitable access to maternal RSV vaccination a public health and ethical imperative. This distinction is particularly relevant in equity analyses, where favorable attitudes may coexist with structural barriers that limit actual vaccine uptake.

Most behavioral and implementation evidence derives from high-income contexts, where studies from Europe and North America consistently document the influence of safety perceptions, prior vaccination behavior, and provider recommendation on intention, preference, and uptake [30,31,32,33]. Canadian and Italian investigations reinforce these patterns [34,35,36].

In contrast, emerging LMIC data remain comparatively sparse. A feasibility study from the Gambia reported generally favorable attitudes toward maternal RSV vaccination but also highlighted concerns related to healthcare access, information provision, and trust in antenatal services [94]. Modelling from South Africa projected substantial mortality reductions, with benefits outweighing potential risks across diverse epidemiological scenarios [6]. Together, these findings suggest that maternal RSV vaccination could meaningfully contribute to child survival strategies in high-burden settings, provided structural and financial barriers are addressed.

Global analyses underscore that equitable deployment requires coordinated policy action. Key barriers include financing constraints, supply limitations, regulatory heterogeneity, cold-chain requirements, and uneven integration into maternal and child health systems [95]. Without pooled procurement strategies, tiered pricing, and strengthened antenatal platforms, new RSV preventive technologies risk widening existing disparities. Early implementation data from high-income countries demonstrate reductions in RSV-associated hospitalizations following maternal vaccination and/or monoclonal antibody rollout [21,22], but achieving similar impact in lower-resource settings will depend on affordability, supply chain reliability, antenatal care coverage, and timely access. Documented cross-national variation in uptake further illustrates the risk of inequitable deployment [51].

Ethically, maternal RSV vaccination requires balancing clear communication of infant benefit with respect for maternal autonomy. Safety concerns during pregnancy remain influential [31,34], making transparent communication of post-marketing surveillance [50], pooled safety analyses [14], and real-world effectiveness evidence [19,20] essential to maintaining trust. In dual-strategy contexts, clear explanation of comparative mechanisms, timing, and duration of protection supports informed decision-making [32,34,52]. Achieving equity therefore requires more than biological efficacy: it demands coordinated financing, strengthened antenatal systems, robust monitoring, and ethically grounded communication strategies that support informed decision-making and facilitate equitable uptake.

## 7. Implications for Clinical Practice and Policy

Effective implementation of maternal RSV vaccination requires coordinated action across behavioral, structural, economic, and health system domains, addressing both determinants of vaccination intention and the factors enabling translation into real-world uptake. Healthcare provider engagement remains foundational, as strong recommendation from obstetricians or midwives consistently increases maternal willingness to vaccinate [30,31,34,36]. Provider training should prioritize clear communication of safety and effectiveness evidence, including real-world protection data [19,20], pooled safety analyses [14], and population-level obstetric outcomes [17,18], ensuring counselling is evidence-based and responsive to maternal concerns. However, provider recommendation alone may be insufficient without enabling structural and system-level conditions.

Embedding RSV vaccination within routine antenatal immunization pathways can reduce logistical barriers and enhance uptake [51]. National recommendations and professional society endorsement—including recent Latin American guidance [27]—strengthen policy coherence and provider confidence. Structural reforms, such as expanding birthing hospital participation in the Vaccines for Children program in the United States, illustrate how financing and delivery mechanisms can promote equitable access [69]. Updated Swedish guidelines further demonstrate alignment between prevention strategies and clinical management pathways [28], underscoring the importance of integration into established maternal–child health infrastructures. Importantly, maternal vaccination and monoclonal antibody prophylaxis are delivered through distinct clinical pathways, requiring coordination across antenatal and neonatal/paediatric services [32,34,51]. These strategies differ in indication, timing, reimbursement mechanisms, and delivery infrastructure [23,24,25,26,66], necessitating tailored implementation approaches rather than a unified delivery model. Collectively, these findings highlight the role of structural determinants in translating favorable maternal attitudes into actual vaccination behavior [39,40,51].

Given the availability of alternative preventive options, counselling must clearly articulate mechanisms, timing, duration of protection, and the complementary roles of maternal vaccination and monoclonal antibodies [32,34,52]. Modelling indicates that population-level impact depends on coverage, timing, and implementation efficiency [23], while cost-effectiveness varies according to pricing and resource constraints across settings [24,25,26]. Regional variation in RSV seasonality necessitates alignment of immunization schedules with local transmission dynamics [91], reinforcing the need for context-specific strategies. Clear communication may support informed decision-making but must be accompanied by accessible delivery pathways to ensure uptake.

Beyond RSV-specific outcomes, maternal vaccination may contribute to broader neonatal infection prevention and antimicrobial stewardship efforts [44], supporting integration within comprehensive maternal–child health strategies.

Robust surveillance systems are essential to monitor coverage, safety, equity indicators, and seasonality alignment during rollout [51]. Strengthened data linkage and real-time monitoring enable early identification of disparities, guide corrective action, and ensure that demonstrated biological efficacy translates into consistent, equitable delivery. Ultimately, successful implementation depends not only on biological performance but on the capacity of health systems to translate intention into sustained, equitable uptake.

## 8. Limitations

This narrative review has several limitations. First, the synthesis reflects substantial heterogeneity in the available evidence base, spanning cross-sectional surveys, qualitative studies, discrete choice experiments, modelling analyses, and observational effectiveness data. Variation in study design, populations, measurement tools, and outcome definitions—particularly the distinction between stated intention, expressed preferences, and documented real-world uptake—limits direct comparability across studies and precludes quantitative aggregation [51]. Conceptual constructs such as risk perception, trust, and safety concerns are operationalized inconsistently, and few studies employ validated behavioral measures, reducing construct validity and limiting the transferability of findings. In addition, the literature search was conducted primarily using PubMed/MEDLINE, supplemented by targeted grey literature searches, and did not include other databases such as Embase. This may have limited the comprehensiveness and reproducibility of study identification.

Second, most behavioral and implementation evidence originates from high-income countries, constraining generalizability to low- and middle-income settings where antenatal care coverage, health-system capacity, financing mechanisms, and cultural norms differ substantially [94,95]. Although modelling studies broaden geographic representation [6], empirical data on program integration, verified coverage, and real-world implementation in high-burden regions remain limited. Structural and cultural determinants—such as medicalization of antenatal care, provider-driven decision-making, and norms surrounding medical interventions during pregnancy—are insufficiently examined across settings.

Third, a substantial proportion of behavioral investigations assess stated vaccination intention rather than documented real-world vaccination behavior, limiting the ability to accurately estimate coverage dynamics and to understand how behavioral determinants translate into actual uptake under routine program conditions. This distinction represents a key limitation of the current evidence base, as intention does not consistently predict observed vaccination behavior. Early implementation data are emerging but remain temporally constrained and subject to reporting variability, registry completeness, and differential access to antenatal services [79].

Fourth, comparative evidence on maternal vaccination and infant monoclonal antibodies remains incomplete. Few studies evaluate real-world preferences, sequencing strategies, or the behavioral and operational implications of dual-strategy availability. As a result, the impact of comparative framing, perceived control, and timing of protection on actual uptake remains insufficiently characterized, particularly with respect to how these factors influence real-world uptake decisions.

Fifth, economic and transmission models rely on assumptions regarding seasonality, duration of protection, baseline disease burden, pricing structures, and achievable coverage levels. These parameters vary across settings and over time, and updates as programs mature may meaningfully alter projected impact and cost-effectiveness estimates [23,90,92]. Incorporating empirically derived uptake parameters, health-system constraints, and context-specific delivery pathways would enhance policy realism.

Finally, few studies apply formal implementation science frameworks such as CFIR, TDF, or RE-AIM. The absence of structured implementation constructs limits the ability to identify modifiable determinants, specify mechanisms of action, and design scalable, context-adapted implementation strategies. Post-marketing surveillance and real-world effectiveness data remain limited given the recency of program introduction, underscoring the need for robust pharmacovigilance, data linkage, and equity-focused monitoring systems.

Despite these limitations, the convergence of biological, clinical, behavioral, economic, and implementation evidence provides a coherent foundation for understanding both determinants of vaccination intention and the structural factors shaping real-world uptake, thereby informing future research and policy deliberation.

## 9. Future Perspectives

Several priority directions emerge for advancing evidence and policy on maternal RSV vaccination. First, prospective longitudinal and registry-linked cohort studies are needed to characterize determinants of documented maternal vaccination behavior and to better understand how stated intention translates into real-world uptake under routine program conditions [51,81]. Linkage between antenatal immunization registries, birth records, and infant outcome datasets would enable accurate assessment of program participation, safety surveillance, and effectiveness across diverse implementation settings [79].

Second, comparative effectiveness and optimization research should continue to evaluate maternal vaccination and monoclonal antibody strategies within dynamic, context-specific frameworks. Modelling consistently shows that projected public health impact and cost-effectiveness are highly sensitive to achievable coverage and seasonal timing [23,91], while additional transmission and optimization models highlight the influence of pricing structures, baseline epidemiology, and program uptake parameters [87,90,93]. Incorporating empirically derived uptake patterns and health-system constraints into economic and transmission models would substantially enhance policy realism, particularly in capturing the gap between projected coverage and observed real-world uptake.

Third, research on communication, risk perception, and decision-making is needed to clarify how evolving safety and real-world effectiveness evidence shapes maternal choices and influences the translation of intention into actual vaccination behavior [31,34]. Experimental and implementation-oriented studies evaluating message framing, provider counselling strategies, and digital information environments are particularly warranted, given evidence that public discourse influences vaccine attitudes during early rollout phases [61].

Fourth, expanding implementation research in low- and middle-income countries is essential. While modelling suggests substantial mortality and morbidity benefits in high-burden settings [6], and feasibility studies indicate general acceptability in African contexts [94], empirical data on health-system integration, financing mechanisms, and verified coverage remain limited. Achieving equitable global impact will require coordinated procurement strategies, context-adapted delivery models, and strengthened antenatal care infrastructure [95].

Finally, sustained pharmacovigilance and real-world effectiveness evaluation will be critical as programs mature. Transparent reporting frameworks, including post-marketing surveillance [2,14,50], are necessary to maintain public confidence, support adaptive policy refinement, and ensure that implementation translates into sustained and equitable uptake.

## 10. Conclusions

Maternal RSV vaccination represents a major advance in early-life infectious disease prevention, supported by strong biological rationale, robust clinical efficacy, and emerging real-world effectiveness. Within the current dual-strategy landscape, maternal vaccination—together with long-acting monoclonal antibodies—offers a realistic opportunity to substantially reduce RSV-associated hospitalization and infant respiratory morbidity, provided that effective implementation translates into high and sustained uptake. Yet biological performance alone cannot secure population-level benefit. A critical challenge lies in bridging the gap between favorable maternal attitudes and actual vaccination behavior under real-world conditions. Sustained and equitable impact depends on high coverage, seamless integration into antenatal care systems, context-sensitive policy implementation, and transparent communication addressing safety, effectiveness, and the comparative roles of available preventive options. By synthesizing behavioral, structural, economic, and policy determinants within a unified analytical framework, this review clarifies both the behavioral determinants of vaccination intention and the structural factors shaping real-world uptake, providing a strategic foundation for implementation research, policy translation, and equitable global deployment.

## Figures and Tables

**Table 1:** Representative Empirical Studies Examining Determinants and Uptake of Maternal RSV vaccination (2023–2026).

Country	Study	Design	Population	Strategy	Outcome	Key Facilitators	Key Barriers	Phase
Greece	Damatopoulou 2024 [30]	Cross-sectional	Pregnant women	Maternal vaccine	Intention	High perceived infant risk; prior antenatal vaccination	Safety uncertainty; limited RSV awareness	Pre-implementation
UK	Broad 2025 [31]	National survey	Pregnant/postpartum	Maternal vaccine	Intention	Provider recommendation; perceived disease severity	Safety concerns in pregnancy; low RSV knowledge	Pre-implementation
Canada	McClymont 2025 [34]	National survey	Pregnant/postpartum	Maternal vs. mAb	Preference	Trust in healthcare providers; perceived infant benefit	Preference shift with safety concerns	Early implementation
USA	Nuzhath 2025 [38]	Cross-sectional	Pregnant women	Maternal vs. mAb	Preference	Direct infant protection framing	Differences in safety perception	Early implementation
Japan	Okubo 2026 [39]	Nationwide survey	Pregnant women	Maternal vaccine	Uptake	Provider engagement; system access	Limited awareness; reimbursement uncertainty	Implementation
USA	Blauvelt 2025 [40]	Multisite cohort	Pregnant women/infants	Maternal mAb	Uptake	Health-system coordination	Coverage disparities	Implementation
France	Bonnel 2025 [41]	Prospective cohort	Infants (maternal/mAb context)	mAb (nirsevimab)	Uptake	Structured campaign rollout	Socioeconomic variability	Implementation
UK	Razai 2025 [42]	Cross-sectional	Pregnant women	Maternal vaccine	Uptake	National recommendation; service integration	Socioeconomic gradients	Implementation

Abbreviations: RSV, respiratory syncytial virus; mAb, monoclonal antibody; UK, United Kingdom; USA, United States.

**Table 2:** Multilevel Determinant Framework for Maternal RSV Vaccination Uptake.

Determinant Level	Determinant Construct	Theoretical Mapping	Empirical Evidence	Direction of Association
Individual	Perceived infant susceptibility/severity	Health Belief Model (Perceived risk)	Damatopoulou 2024 [30]; Callaghan 2025 [32]	↑ intention
Individual	Vaccine safety confidence	5C—Confidence	Broad 2025 [31]; McClymont 2025 [34]	↓ acceptance when low
Individual	Comparative decision framing (maternal vs. mAb)	Risk–benefit appraisal; decisional balance	Callaghan 2025 [32]; McClymont 2025 [34]; Nuzhath 2025 [38]	Preference shift when maternal safety concerns present
Interpersonal	Provider recommendation	Social norms; cue to action (HBM)	Broad 2025 [31]; McClymont 2025 [34]	↑ uptake
Structural	Reimbursement and delivery integration	5C—Constraints	Trusinska 2025 [51]; Okubo 2026 [39]	↑ coverage when funded and integrated
Structural	Equity and access constraints	Access theory; health equity frameworks	Blauvelt 2025 [40]; Bonnel 2025 [41]	↓ uptake in socioeconomically disadvantaged groups
Policy	National endorsement and guideline alignment	Institutional trust; policy diffusion	Montesinos 2026 [27]; Navér 2026 [28]	↑ system integration and provider confidence

Abbreviations: RSV, respiratory syncytial virus; HBM, Health Belief Model; 5C, confidence, complacency, constraints, calculation, collective responsibility. This arrow “↓” means lower and that arrow “↑” means higher.

**Table 3:** Comparative Characteristics of Maternal Vaccination and Infant Monoclonal Antibody Strategies.

Dimension	Maternal Vaccination	Infant mAb (e.g., nirsevimab)
Timing of administration	During late pregnancy (antenatal period)	At birth or early infancy
Mechanism of protection	Transplacental transfer of vaccine-induced maternal IgG antibodies	Direct passive administration of monoclonal antibodies to the infant
Biological dependency	Requires adequate maternal immune response and placental transfer	Independent of maternal immune status
Maternal exposure	Yes (maternal systemic immune activation)	No maternal exposure
Onset of infant protection	Immediately at birth (if administered within recommended gestational window)	After infant administration
Duration of protection	Limited to early infancy; dependent on antibody waning kinetics	Defined duration based on monoclonal antibody half-life
Primary behavioural driver	Maternal risk–benefit evaluation during pregnancy	Preference for direct infant-targeted protection
Key safety perception focus	Vaccine safety during pregnancy	Infant safety and novelty of biologic agent
Structural integration pathway	Integrated into routine antenatal care services	Delivered through neonatal or paediatric services
Primary delivery setting	Obstetric/antenatal care services	Neonatal units/paediatric clinics/birth hospitals
Reimbursement pathway	Antenatal immunization programs/maternal health budgets	Infant immunization programs/paediatric or hospital-based funding mechanisms
Policy implementation considerations	Requires antenatal coverage and provider recommendation	Requires procurement, cold-chain logistics, and infant follow-up systems

Abbreviations: mAb, monoclonal antibody; IgG, immunoglobulin G.

## Data Availability

No new data were created or analyzed in this study. Data sharing is not applicable to this article.

## Supplementary Materials

The following supporting information can be downloaded at: https://www.mdpi.com/article/10.3390/vaccines14040293/s1, Supplementary Table S1. Detailed Search Strategy and Eligibility Criteria; Supplementary Table S2. Characteristics of Included Empirical Studies Examining Determinants and Uptake of Maternal RSV vaccination (2023–2026).
