# Supplementary material for: Determinants of Maternal RSV Vaccination Uptake: A Narrative Review
Source: Vaccines (Basel). 2026 Mar 26;14(4):293. doi: 10.3390/vaccines14040293 (PMC13119929; doi:10.3390/vaccines14040293)
Supplement: Supplementary file 1 [file vaccines-14-00293-s001.zip › vaccines-4211761-supplementary.pdf]

Supplementary Table S1. Detailed Search Strategy and Eligibility Criteria

Section	Details
Database: PubMed/MEDLINE	("respiratory syncytial virus" OR RSV) AND ("maternal vaccination" OR pregnancy OR antenatal OR "maternal immunization") AND (uptake OR acceptance OR intention OR hesitancy OR determinants OR coverage OR implementation) AND ("monoclonal antibodies" OR nirsevimab OR immunoprophylaxis)
Database: Google Scholar	respiratory syncytial virus maternal vaccination uptake determinants monoclonal antibodies nirsevimab
Date Range	January 2022 – February 2026
Language	English
Population	Human studies
Study Types	Observational studies, surveys, qualitative studies, implementation studies, modelling studies, policy reports
Inclusion Criteria	Studies examining maternal RSV vaccination uptake, acceptance, or intention; determinants (behavioral, interpersonal, structural, economic, policy); parental preferences; real-world implementation; economic or modelling analyses; policy-relevant insights
Exclusion Criteria	Non-RSV studies; preclinical or immunogenicity-only studies; studies without uptake/implementation relevance; commentaries without empirical data (unless policy-relevant); duplicates

Notes	Combination of controlled vocabulary and free-text terms; Boolean operators (AND/OR); database-specific adaptations; grey literature from public health organizations (e.g., WHO)

Supplementary Table S2. Characteristics of Included Empirical Studies Examining Determinants and Uptake of Maternal RSV vaccination (2023–2026)

First Author (Year)	Country/Setting	Study Design	Population	Key Findings
Wang (2025) [24]	Hong Kong	Economic model	Population-level	Maternal vaccination cost-effective under certain assumptions
Rave (2025) [25]	Nepal	Economic model	Population-level	Context-specific cost-effectiveness
Taleshi (2025) [28]	Canada	Economic model	Population-level	Program impact depends on coverage and cost
Callaghan (2025) [32]	USA/Italy	Survey	Pregnant women	Disease severity perception increases willingness
Gidengil (2023) [33]	USA	Survey	Pregnant women	Positive vaccine attitudes associated with higher acceptance
McClymont (2025) [34]	Canada	National survey	Pregnant/postpartum	77% maternal vaccine acceptance; preference shifts with safety concerns

**First Author (Year)**	**Country/Setting**	**Study Design**	**Population**	**Key Findings**
Miraglia Del Giudice (2023) [35]	Italy	Survey	Pregnant women	Moderate willingness; influenced by knowledge and risk perception
Gagnon (2025) [36]	Canada	Mixed-methods	Pregnant women	Provider recommendation and trust key drivers
Huang (2026) [37]	Taiwan	Survey	Parents	Knowledge and perceived risk influence acceptance
Trusinska (2025) [46]	Global	Systematic review	Mixed	Uptake varies widely across systems and policies
Langedijk (2025) [47]	Netherlands	Discrete choice	Parents	Cost, convenience, and delivery setting influence decisions
Machida (2025) [52]	Japan	Survey	Pregnant women	Knowledge correlates with positive attitudes
Demirci (2025) [53]	Turkey	Survey	Pregnant women	Awareness linked to favorable vaccination views
Paulson (2025) [58]	UK	Survey	Parents	Safety perception and provider trust shape preferences
Nuzhath (2025) [59]	USA	Cross-sectional	Pregnant women	Preferences influenced by framing and perceived infant protection
Kuntz (2025) [60]	USA	Survey	Parents	Knowledge and safety perceptions influence acceptance

**First Author (Year)**	**Country/Setting**	**Study Design**	**Population**	**Key Findings**
Felek Boyvat (2025) [61]	USA	Qualitative	Immigrant families	Cultural and communication factors affect decisions
Okubo (2026) [66]	Japan	Nationwide survey	Pregnant women	Uptake influenced by access, awareness, and provider engagement
Oka (2025) [67]	Japan	Qualitative	Pregnant women	Barriers include limited awareness and policy ambiguity
Blauvelt (2025) [68]	USA	Multisite cohort	Pregnant women/infants	Uptake 64%; disparities observed; high combined protection
Bonnel (2025) [71]	France	Cohort	Infants	Uptake influenced by socioeconomic factors
Razai (2025) [72]	UK	Cross-sectional	Pregnant women	Uptake linked to national policy and integration
Boundy (2025) [73]	USA	Surveillance	Infants	Coverage expanding but incomplete
Irving (2025) [75]	USA	Registry study	Infants	Variability in coverage across systems
Homo (2026) [76]	USA	Observational	Neonates	Uptake associated with demographic factors
Höck (2025) [77]	Austria	Observational	Infants	Uptake associated with reduced RSV burden
Ghirardo (2026) [78]	Italy	Multicenter study	Infants	Reduction in RSV hospitalizations post-implementation
Bugden (2025) [87]	Canada	Economic model	Population-level	Comparative cost-effectiveness of strategies

**First Author (Year)**	**Country/Setting**	**Study Design**	**Population**	**Key Findings**
Averin (2025) [88]	USA	Economic model	Population-level	Cost-effectiveness sensitive to coverage and price
Zhu (2025) [90]	Global	Systematic review	Mixed	High heterogeneity in economic evaluations
Jasseh (2025) [94]	Gambia	Feasibility study	Pregnant women	Acceptability high but access barriers remain
